# Cellular immunotherapy as maintenance therapy prolongs the survival of the patients with small cell lung cancer

**DOI:** 10.1186/s12967-015-0514-0

**Published:** 2015-05-13

**Authors:** Xiao Ding, He Cao, Xiao Chen, Haofan Jin, Ziling Liu, Guanjun Wang, Lu Cai, Dan Li, Chao Niu, Huimin Tian, Lei Yang, Yuguang Zhao, Wei Li, Jiuwei Cui

**Affiliations:** Cancer Center, the First Hospital of Jilin University, No. 71. Xinmin Street, Changchun, 130021 China; Kosair Children’s Hospital Research Institute, Department of Pediatrics, the University of Louisville, Louisville, KY 40202 USA

**Keywords:** Small cell lung cancer, Cellular immunotherapy, Maintenance therapy

## Abstract

**Background:**

Small cell lung cancer (SCLC) relapses rapidly after the initial response to chemotherapy and shows drug-resistance. This study was to investigate the efficacy and safety of cellular immunotherapy (CIT) with autologous natural killer (NK), γδT, and cytokine-induced killer (CIK) cells as maintenance therapy for SCLC patients.

**Methods:**

A pilot prospective cohort study was conducted with SCLC patients who had responded to initial chemotherapy. Patients elected to receive either CIT as maintenance therapy (study group), or to be followed-up without further treatment (control group). Progression-free survival (PFS), overall survival (OS), and adverse effects were investigated.

**Results:**

We recruited 58 patients (29 in each group). The patient characteristics of the 2 groups were well balanced. PFS was not significantly different between the groups, but OS was significantly longer in the study group than the control (20 vs. 11.5 months, P = 0.005; hazard ratio [HR], 0.434, 95 % confidence interval [CI], 0.236–0.797, P = 0.007). Among patients with limited-stage disease, there was no difference in PFS between the groups, but OS was longer in the study group compared to the control (26.5 vs. 11.8 months, P = 0.033; HR, 0.405, 95 % CI, 0.169–0.972, P = 0.043). Among patients with extensive-stage disease, both PFS and OS were longer in the study group than the control (5 vs. 2.7 months, P = 0.037; HR, 0.403, 95 % CI, 0.162–1.003, P = 0.051, and 14.5 vs. 9 months, P = 0.038; HR, 0.403, 95 % CI, 0.165–0.987, P = 0.047, respectively). No significant adverse reactions occurred in patients undergoing CIT.

**Conclusions:**

CIT maintenance therapy in SCLC prolonged survival with only minimal side effects. Integrating CIT into current treatment may be a novel strategy for SCLC therapy, although further multi-center randomized studies are needed.

**Electronic supplementary material:**

The online version of this article (doi:10.1186/s12967-015-0514-0) contains supplementary material, which is available to authorized users.

## Background

Lung cancer is the most commonly diagnosed cancer and also the leading cause of cancer death globally [1]. Small cell lung cancer (SCLC) is usually reported to comprise about 15 % of all lung cancer cases recorded [2]. Despite a high initial response rate to first-line therapy, most patients die rapidly from recurrent, drug-resistant disease. Even with more advanced chemotherapeutic agents and molecularly targeted drugs, the prognosis of this disease remains poor due to limited treatment efficacy [3–5]. Recently, maintenance therapy in advanced non-small cell lung cancer (NSCLC) has been found to be an acceptable treatment paradigm to improve progression free survival (PFS) [6]. However, a meta-analysis of published randomized clinical trials [7] showed that both maintenance and consolidation therapy failed to improve the outcomes of SCLC, and in some cases even caused severe side effects or toxic death. Thus, there is no recommendation for maintenance therapy in current SCLC treatment guidelines. Given its high recurrence rate and mortality, new therapeutic strategies are urgently needed to improve the outcome of this disease.

Immune escape plays an important role in cancer recurrence and metastasis [8, 9]. With an improved mechanistic understanding of immune response and immune escape, several immunotherapies were investigated in SCLC. Some of them were failed, such as the dendritic cell-based p53 vaccine [10], but some of them obtained a certain effect, such as phased ipilimumab (an antibody against cytotoxic T-lymphocyte antigen-4 [CTLA-4]) with paclitaxel/carboplatin exhibited improved immune-related PFS (irPFS) [11]. It indicated that immunotherapy might have the potential to improve the prognosis of SCLC. Besides, it also suggested that different patterns of immunotherapy combined with chemotherapy might have an influence on the prognosis of SCLC. Therefore, increasing attention has been paid to the possibility of immunotherapy for SCLC patients in recent years.

SCLC patients have often been found to have a functional deficiency in a variety of immunocytes [12–14], implying that adoptive transfusion of *ex vivo-*activated and expanded immunocytes may be a feasible and effective therapy. Cellular immunotherapy (CIT) has been shown to be effective for a variety of cancers [15–17], but its use in SCLC patients has not been reported.

Activated immune cells can reach the lungs within minutes of intravenous injection and selectively enter malignant tissue. Consequently, a substantial number of these cells can accumulate at cancer sites within 24 hr of treatment [18, 19]. We postulated that CIT would provide an anti-caner effect and prolong the survival of SCLC patients. However, it is not yet known which cell types are needed in CIT for performing anti-cancer effects optimally.

Several specific immunotherapies to induce cytotoxic T lymphocyte (CTL) for SCLC have been tried, such as the dendritic cell-based p53 vaccine [10], few of them have lengthened survival, partly due to the complexity of the immune escape mechanism in this malignancy. Decreased expression of HLA-class I antigen has been reported in SCLC, which may be one of the mechanisms of SCLC cells to escape CTL attack [20]. Natural killer (NK) and γδT cells are effector cells of innate immunity, and both can exert anti-cancer effects in a non-MHC-restricted manner. Cytokine-induced killer (CIK) cells are *ex vivo*-activated lymphocytes, and represent a heterogeneous cell population, including CD3^+^CD56^+^, CD3^+^CD56^−^ (typical T) and CD3^−^CD56^+^ (NK) cells. The anti-cancer activity of CIK cells is mainly due to the CD3^+^CD56^+^ cells, which show an NK-like, non-MHC-restricted cytolytic activity against cancer cells. CIT based on any of these cell types has proved to be effective against a variety of cancers [15–17]. SCLC cells were also found to be susceptible to NK or γδT cell-mediated cytotoxicity in preclinical studies [20, 21]. In addition, γδT cells can induce the NK cell-mediated killing of cancers that are usually resistant to NK cytolysis [22] and cross-present tumor antigens to CD8^+^ CTLs to mediate adaptive immune responses [23]. These findings suggest that the combined application of NK, γδT and CIK cells may provide significantly synergistic anti-cancer effects via different mechanisms, and could provide effective CIT for SCLC patients. We therefore conducted this pilot prospective cohort study to evaluate the efficacy and safety of combined NK, γδT, and CIK cell based CIT as a maintenance therapy for SCLC patients, with the objective of consolidating remission and prolonging survival in patients who responded to first-line therapy.

## Methods

### Patients and study design

A pilot prospective cohort study was conducted to evaluate the efficacy and safety of combined NK, γδT, and CIK cell based CIT as a maintenance therapy for SCLC patients. All patients with SCLC that met the following criteria at the First Hospital of Jilin University were included in this study after June 1, 2009.

Eligibility: Patients had to (i) have been diagnosed as SCLC and have completed first-line therapy, (ii) have achieved stable disease (SD), partial remission (PR) or complete remission (CR) after the first-line treatment, (iii) be at least 18 years old, (iv) have an Eastern Cooperative Oncology Group (ECOG) performance status ≤ 2, (v) have normal kidney, liver, and bone marrow function and be free of cardiac arrhythmias, congestive heart failure or severe coronary artery disease, and (vi) have a life expectancy ≥ 3 months. Exclusion criteria included (i) autoimmune disease, (ii) a clinically serious infection, (iii) for women, pregnancy or lactation, (iv) a history of organ transplantation, and (v) the administration of another immunotherapy. This study was conducted in accordance with the Declaration of Helsinki and was reviewed and approved by the Ethical Committee of the First Hospital of Jilin University [ID #: 2009–020]. Written informed consent was obtained from all patients before their enrollment into the study.

Treatment plan: The treatment regimens of SCLC patients in this study were based on the Small Cell Lung Cancer NCCN Guidelines – version 2.2009 [24]. The first-line therapy regimen for limited stage SCLC (LS-SCLC) patients was platinum based chemotherapy, e.g.: etoposide/cisplatin (EP) regimen or etoposide/carboplatin (EC) regimen for 4–6 cycles plus concurrent chest radiotherapy (1.8–2 Gy once daily to 60–70 Gy) [24]. The initial chemotherapy for extensive stage SCLC (ES-SCLC) patients was the same as those for LS-SCLC patients.

**Fig. 1:**
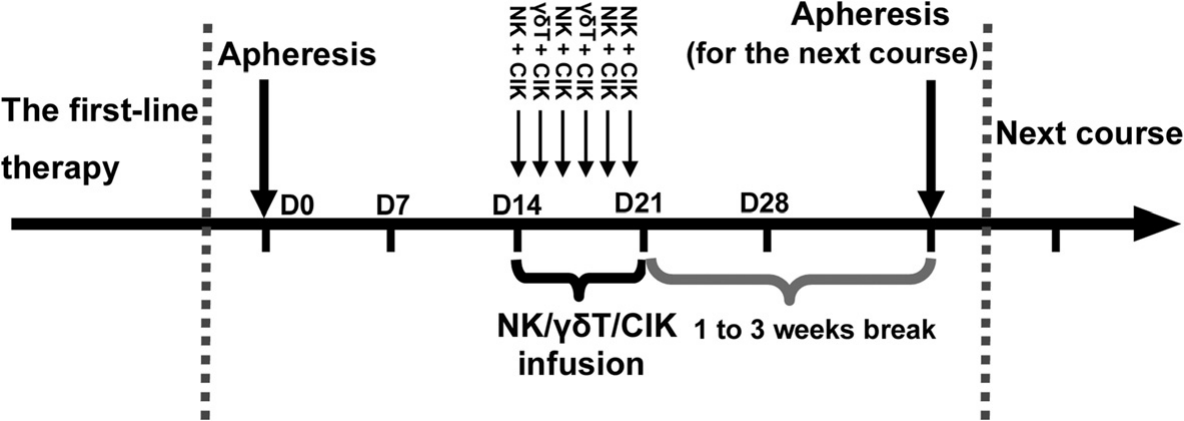
The schedule for autologous cellular immunotherapy (CIT). Autologous peripheral blood mononuclear cells (PBMCs) were collected by apheresis on D0, and were induced into NK, γδT, or CIK cells. The expanded immunocytes were then infused back into patients 14 days later (D14) as the initial transfusion. There were for 6 consecutive transfusion days (D14–D20) with 2 kinds of immune cells for each infusion, and each CIT course was completed within 3 weeks of apheresis. The second course of PBMCs collection was started 1 to 3 weeks after the end of the first course

After first-line therapy, patients either received CIT (at least 1 course) as maintenance treatment (study group), or were followed up without further treatment (control group). The decision whether to undergo CIT or to be entered into the control group was made by the patient. For the study group, autologous peripheral blood mononuclear cells (PBMCs) were collected by apheresis on D0, and were induced into NK, γδT, or CIK cells. The expanded immunocytes were then infused back into the patients 14 days later (D14) as the initial transfusion. There were for 6 consecutive transfusion days (D14–D20) with 2 kinds of immune cells for each infusion, and each CIT course was completed within 3 weeks after apheresis. The second course of PBMCs collection was started 1 to 3 weeks after the end of the first course. The treatment schedule is summarized in Fig. 1. Maintenance treatment was continued unless there was progressive disease (PD) or the patient refused to undergo further CIT. In cases of PD, the patient was given either a best supportive treatment or a second or even a third-line chemotherapy regimen, depending on their general health status and/or preference.

The second-line chemotherapy regimens were selected based on the time of relapse. If patients experienced relapse within 6 months after the first-line therapy, patients would be treated with topotecan or irinotecan; if patients relapsed more than 6 months after the completion of first-line therapy, they would receive the original regimen. The third-line chemotherapy regimens were selected based on the previous chemotherapy. Patients could be given irinotecan, topotecan or paclitaxel that had not been used in the previous chemotherapy.

Follow up: In general, follow-up was required every 3 months. Each follow-up included a complete physical examination, basic serum chemistry, and computed tomography (CT) of the chest and abdomen. Brain magnetic resonance imaging (MRI) and a technetium bone scan were performed as clinically indicated.

### Preparation of immune cells

All procedures for preparing the autologous immune cells were carried out under Good Manufacturing Practice (GMP) conditions (Certificate ID: A20090047) which were approved by the Jilin Provincial Center for Sanitation Inspection and Test. The preparation of and quality control for each type of immunocytes was performed in strict accordance with the standard operating procedure (SOP). Immune cells were prepared as described in our previous study [25], with some modifications based upon previously published procedures [26, 27]. Briefly, about 1.5 × 10^9^ (1.0–2.0 × 10^9^) PBMCs were collected from the patient using a Cobe Spectra Apheresis System (Gambro BCT, Inc. USA). These PBMCs were separated into 2 parts and placed in 50 ml centrifuge tubes that were centrifuged for 10 min at 3000 rpm. The supernatant was then removed, and the cell pellets were re-suspended in 30 ml PBS, and placed on top of a 15 ml Ficoll-Hypaque (Amersham Biosciences, Uppsala, Sweden) in a sterile 50 ml tube. Lymphocytes were isolated from PBMCs using Ficoll-Hypaque density centrifugation (Ficoll separation). Approximately 1.3 × 10^9^ (1.0 − 1.8 × 10^9^) mononuclear cells were obtained after these procedures. The cells were then separated into 3 pools to induce NK, γδT and CIK cells using different cytokines.

For expansion of NK cells, PBMCs were cultured in AIM-V medium (Invitrogen, Carlsbad, CA, USA), 700 U/mL IL-2 (Miltenyi, Cologne, German) and 1 ng/mL OK432 (Shandong Lu Ya Pharmaceutical, Jining, China) for 24 hr at 37 °C, in a mouse anti-human CD16 monoclonal antibody (mAb; Beckman Coulter, Marseille, France) coated flask. The cultured cells were then centrifuged, and the supernatant was discarded. The cells were again cultured in AIM-V medium and 700 U/mL IL-2 at 37 °C for 2–3 weeks. IL-15 (Miltenyi, Cologne, Germany) was added to the culture medium to promote the growth of NK cells [25, 26]. To generate γδT cells, PBMCs were stimulated with 1 μM zoledronate (Zometa®, Novartis, Beijing, China) in AIM-V medium containing 400 U/mL human IL-2. Fresh medium and IL-2 supplement at 400 U/mL were added every 3 days [25, 27]. To prepare CIK cells, PBMCs were cultured in AIM-V medium and 1000 U/mL IFN-γ (Miltenyi, Cologne, German) at 37 °C for 24 hr. Then, 100 ng/mL mouse anti-human CD3 mAb (Peprotech, Rocky Hill, NJ, USA), 1000 U/mL IL-2 and 1000 U/mL IL-1α (Miltenyi, Cologne, German) were added to the media. Fresh complete medium and IL-2 supplement at 1000 U/mL were added every 3 days [25].

Before transfusion, a fraction of cells was collected so that they could be enumerated, evaluated for viability and phenotype, and checked for possible contamination.

### Phenotypic analysis of immunocytes before infusion

The phenotype of immunocytes were determined on the basis of their specific cell surface markers using 4-color flow cytometry performed on a FACSCalibur (BD Biosciences, San Diego, CA, USA) with directly conjugated mAbs against the markers. Briefly, cultured NK cells were collected, washed, and incubated with mouse mAbs against human CD3-PerCP, CD69-PE, and CD56-APC (BD Biosciences) for 15 min. The γδT cells were incubated with Vγ9-FITC and CD3-APC (BD Biosciences), and the CIK cells were incubated with CD3-PerCP, CD4-FITC, CD8-PE and CD56-APC (BD Biosciences). Isotype-matched antibodies were used as controls.

### Administration of immune cells

The dye-exclusion test was used to assess the viability of the final cell suspension. Possible contamination of the immune cells was tested using a PCR-based assay for mycoplasma, as well as assays for endotoxins, bacteria, and fungi, 24 hr before and on the day of product release. The immunocytes could not be used for patients if they failed to meet the following release criteria: (i) a viability of more than 95 %, (ii) no contamination by bacteria, fungi, endotoxins, or mycoplasma in either of the 2 assessments, (iii) at least 1.2 − 2.0 × 10^9^ of each type of cell per infusion; and (iv) more than 50 % of the cells had the NK (CD3^−^CD56^+^) or γδT (CD3^+^Vγ9^+^) phenotype, and more than 20 % of cells had the CD3^+^CD56^+^ CIK phenotype in NK, γδT and CIK cell culture system respectively, as detected by flow cytometry. Before reinfusion, immunocytes were washed 3 times with normal saline and re-suspended in 50 mL of normal saline. The cells were then administered to patients via an intravenous drip in 30 min. The number of cells in each transfusion ranged from 2.4 − 4.0 × 10^9^.

### Cytotoxic effects of immune cells *in vitro*

The cytotoxicity of immune cells was examined by measuring lactate dehydrogenase (LDH) levels in the culture medium, according to the manufacturer’s instructions for the CytoTox 96 Non-Radioactive Cytotoxicity Assay Kit (Promega, Madison, WI). The target cells used for this assay were the SCLC-derived cell line NCI-H446, and 5 × 10^4^ cells were used to examine the cytotoxic effects of immune cells *in vitro*. The tested ratios of effector cells to target cells (E/T) were 25:1, 12:1, 6:1, and 3:1. In order to investigate the synergistic effect of the different kinds of the immune cells, we combined the 3 types of immunocytes at a ratio of 1:1:1 to examine their cytotoxic effect on target cells.

### Clinical assessment

Overall survival (OS) was the primary end point, and the secondary end points included PFS, safety of CIT and clinical benefit rate (CBR) of the second-line chemotherapy. Response was determined based on the National Cancer Institute’s Response Evaluation Criteria in Solid Tumors (RECIST 1.1) guidelines [28]. OS was defined as the period from the day on which first-line treatment was completed to death from any cause. PFS was defined as the period between the completion of first-line treatment and the onset of PD or death from any cause. CBR was defined as the percentage of patients with CR + PR + SD.

### Proportion of the immune cells in peripheral blood

In order to detect the alteration of the proportion of the immune cells of the patients, two milliliters of peripheral blood was collected before the apheresis and 1 week after the end of one CIT course. T cells (CD3^+^CD4^+^, CD3^+^CD8^+^), NK cells (CD3^−^CD56^+^), NKT cells (CD3^+^CD56^+^), B cells (CD19^+^), regulatory T (Treg) cells (CD4^+^CD25^+^Foxp3^+^) and monocytes (CD14^+^) populations were analyzed with FACSCalibur (BD Biosciences). All antibodies were purchased from BD Biosciences.

### Statistical methods

All calculations were performed using SPSS 17.0 software (SPSS, Chicago, IL). PFS and OS were assessed according to the Kaplan-Meier method and compared between groups using the log-rank test. The multivariate Cox proportional hazard model was applied to analyze factors found to be statistically significant by univariate analysis. The Mann–Whitney test was used to compare medians, and Fisher’s exact test was used to compare binary outcomes. P ≤ 0.05 was considered to be statistically significant.

## Results

### Patient characteristics

A total of 58 eligible patients were recruited between June 1, 2009 and November 1, 2012, with 29 patients in each group. Follow-up of all patients was ended on December 30, 2013, with a median follow-up time of 13 months (range, 3–54 months). Of the 29 patients in each group, 17 patients had LS-SCLC and 12 had ES-SCLC. Two ES-SCLC patients in CIT group had one solitary pulmonary lesion highly suspected as lung cancer under the CT scan. Their abdominal CT and brain MRI were normal. They underwent surgery after these examinations, and pathologically confirmed SCLC. Considering the properties of invasion and metastasis of SCLC, technetium bone scan was performed after surgery for clinical stage. Bone scan showed that one patient had lumbar metastasis, and the other patient had pelvic metastasis. Therefore, they were diagnosed as ES-SCLC. The patient demographics were well balanced between the groups, including characteristics such as sex, smoking index, ECOG performance status, chemotherapy courses, chemotherapy responses, radiotherapy and surgery (Table 1). The only exception was age, as ES-SCLC patients in the CIT group were on average older than those in the control group (P < 0.05).

**Table 1:** Clinical characteristics of patients in the treatment and control groups

Clinical features	LS-SCLC		P value	ES-SCLC		P value	All patients		P value
	Control	Study		Control	Study		Control	Study	
Sex									
Male	14	11	0.438	7	11	0.155	21	22	1.000
Female	3	6		5	1		8	7	
Age, years									
Median	61	61	0.898	58.5	62.5	0.045	60	62	0.215
Range	43–80	41–77		46–67	54–79		43–80	41–79	
Smoking index									
Median	400	300	0.572	600	600	0.861	500	400	0.716
Range	0–1350	0–2000		0–1200	0–1200		0–1350	0–2000	
ECOG									
≤1	14	15	1.000	12	10	0.478	26	25	1.000
2	3	2		0	2		3	4	
Chemotherapy courses									
Median	6	5	0.141	6	6	1.000	6	6	0.187
Range	4–6	4–6		4–6	4–6		4–6	4–6	
Radiotherapy									
Yes	17	17	1.000	6	8	0.680	23	25	0.730
No	0	0		6	4		6	4	
Surgery									
Yes	2	2	1.000	0	2	0.478	2	4	0.666
No	15	15		12	10		27	25	
Chemotherapy responses *									
CR	7	5	0.822	1	2	1.000	8	7	1.000
PR	7	8		7	6		14	14	
SD	3	4		4	4		7	8	

Abbreviations: ECOG, Eastern Cooperative Oncology Group; CR, complete remission; PR, partial remission; SD, stable disease; LS-SCLC, limited stage small cell lung cancer; ES-SCLC, extensive stage small cell lung cancer

Note: *The responses for the first-line therapy

### Quality of the cultured immune cells

**Fig. 2:**
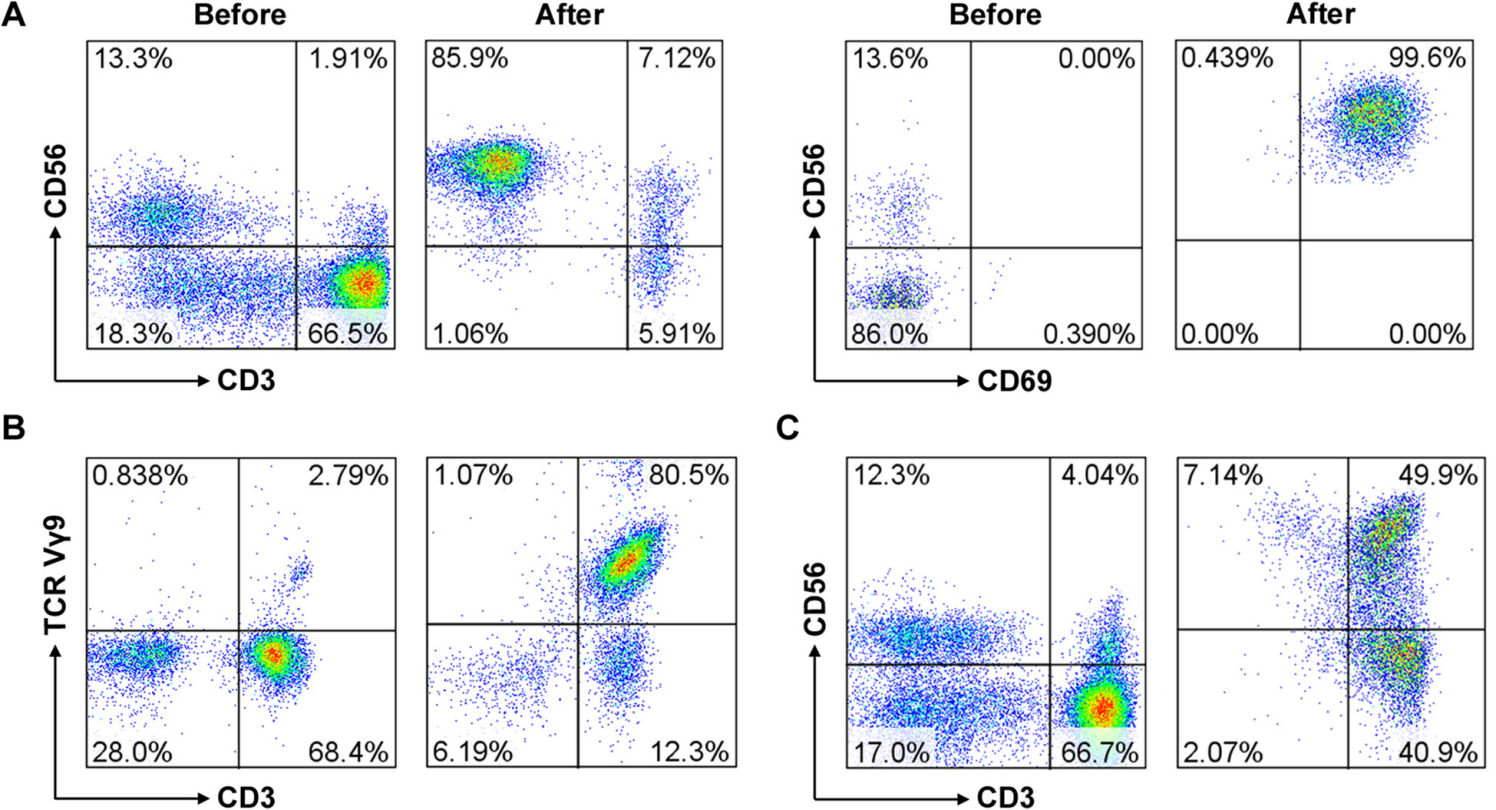
The percentage of NK, γδT and CIK cells before and after induction. Representative results from a single patient are shown. The percentage of NK cells (**A**), γδT cells (**B**) and CIK cells (**C**) before and after induction was 13.3 % vs. 85.9 %, 2.79 % vs. 80.5 % and 4.04 % vs. 49.9 %, respectively. CD56^+^CD69^+^ cells were considered to be activated NK cells

The viability of each type of immune cell in our culture system was found to exceed 95 %, none of the cultured immune cells were found to be contaminated, and all of the preparations met the release criteria. The percentage of NK (CD3^−^CD56^+^), γδT (CD3^+^Vγ9^+^) and CIK (CD3^+^CD56^+^) cells before and after induction was 8.01 % (range, 4.12–17.35 %) vs. 85.32 % (range, 61.33–99.61 %), 4.22 % (range, 2.79–11.26 %) vs. 82.63 % (range, 63.72–98.21 %) and 4.51 % (range, 1.62–7.96 %) vs. 34.52 % (range, 27.25–57.28 %), respectively (Table 2). Thus, each of these cell types was significantly more prevalent after induction. Induction also resulted in a significant increase in the proportion of activated NK cells (CD56^+^CD69^+^). Representative results from a single patient are shown in Fig. 2, and the numbers of the 3 types of immunocytes infused into each patient are shown in Additional file 1: Table S1.

**Table 2:** Summarized data of the percentage of NK, γδT and CIK cells before and after induction

Immune cells	Before (median, range)	After (median, range)
NK cells	8.01 % (4.12–17.35 %)	85.32 % (61.33–99.61 %)
γδT cells	4.22 % (2.79–11.26 %)	82.63 % (63.72–98.21 %)
CIK cells	4.51 % (1.62–7.96 %)	34.52 % (27.25–57.28 %)

Abbreviations: NK, natural killer; CIK, cytokine-induced killer

### Cytotoxic effect of immune cells on cancer cells

The cytotoxic effect of immune cells was examined by measuring LDH release levels. All 3 types of immune cells exhibited a significant cytotoxic effect on NCI-H446 cells when used alone, but a greater cytotoxic effect was achieved when they were combined, and this cytotoxicity increased further as the E/T cell ratio increased. At an E/T cell ratio of 25:1, the median cytotoxicity level of NK, γδT and CIK cells was 75.5 % (range, 59.1–90.7 %), 70.6 % (range, 48.3–80.0 %), and 49.0 % (range, 27.4–68.9 %), respectively. The combined immune cells showed a synergistic anti-cancer effect with a median cytotoxicity level of 85.5 % (range, 63.4–96.5 %) at an E/T cell ratio of 25:1 (Additional file 2: Figure S1).

### PFS and OS

**Fig. 3:**
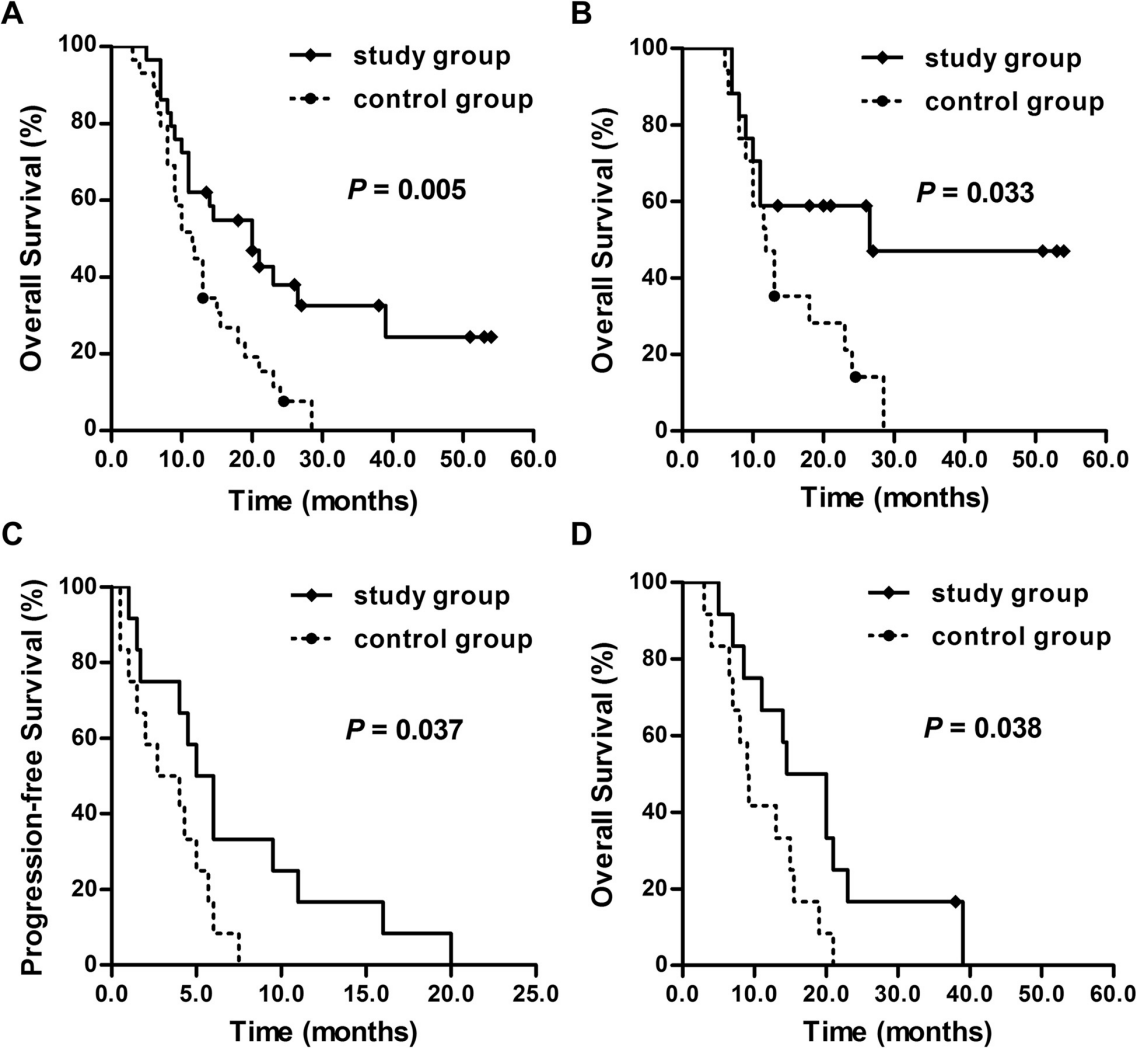
Progression free survival (PFS) and overall survival (OS) in both groups. (**A**) OS of all patients. The OS in the study group was significantly longer than that in the control group (20 vs. 11.5 months; P = 0.005). (**B**) OS of the limited-stage patients. OS in the study group was significantly longer than in the control group (26.5 vs. 11.8 months; P = 0.033). (**C**) PFS of the extensive-stage patients. PFS in the study group was longer than the control group (5 vs. 2.7 months; P = 0.037). (**D**) OS of the extensive-stage patients. OS in the study group was significantly longer than in the control group (14.5 vs. 9 months; P = 0.038)

The median PFS did not differ significantly between the groups (hazard ratio [HR], 0.667; 95 % confidence interval [CI], 0.380–1.169; P = 0.157). However, the median OS in the study group was significantly longer than that in the control group (20 vs. 11.5 months, P = 0.005; HR, 0.434, 95 % CI, 0.236–0.797, P = 0.007) (Fig. 3A).

Patients were divided into two subgroups according to disease stage: LS-SCLC and ES-SCLC. Among the former, there was no significant difference in PFS (HR, 0.751; 95 % CI, 0.349–1.618; P = 0.465); however, the OS of the study group was longer than that of the control group (26.5 vs. 11.8 months, P = 0.033; HR, 0.405, 95 % CI, 0.169–0.972, P = 0.043) (Fig. 3B). Among the ES-SCLC patients, both the PFS and OS of the study group were longer than those of the control group (5 vs. 2.7 months, P = 0.037; HR, 0.403, 95 % CI, 0.162–1.003, P = 0.051, and 14.5 vs. 9 months, P = 0.038; HR, 0.403, 95 % CI, 0.165–0.987, P = 0.047, respectively) (Fig. 3C, D).

### Potential factors influencing the outcome of CIT

**Fig. 4:**
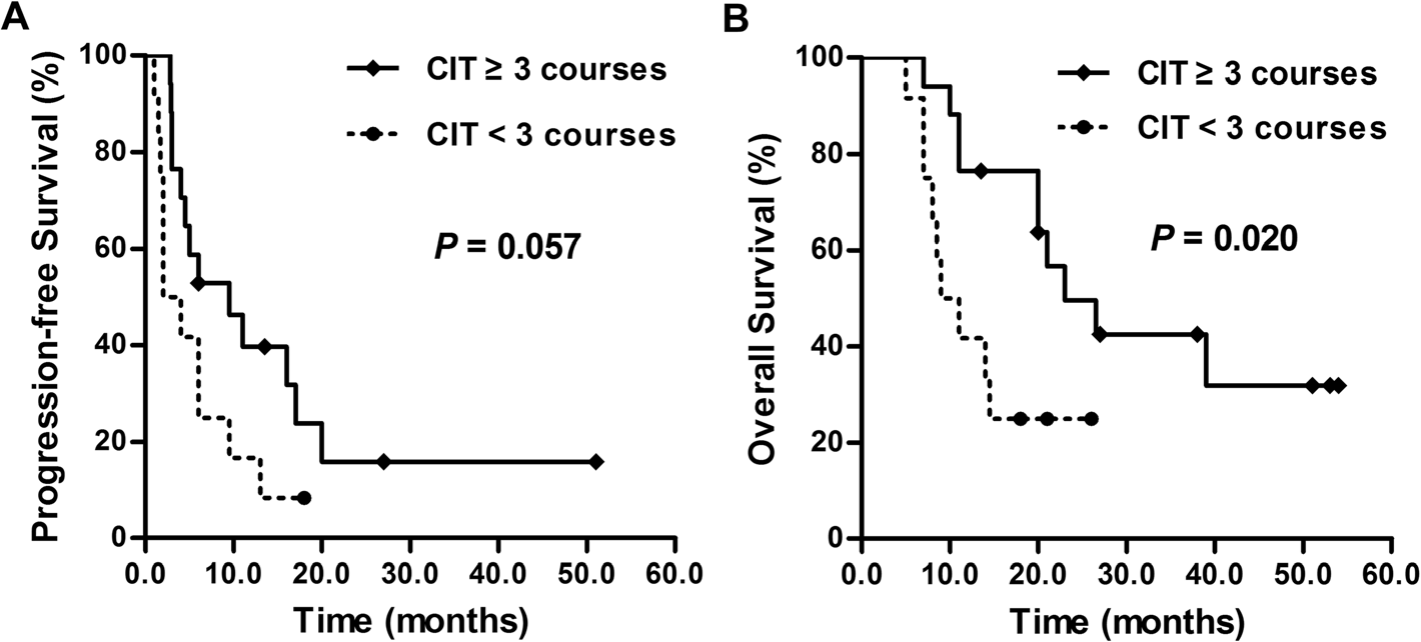
The influence of cellular immunotherapy (CIT) courses on small cell lung cancer (SCLC) patients’ prognosis. (**A**) The median progression free survival (PFS) in the CIT ≥ 3 courses group was longer than that of the CIT < 3 courses group (9.5 vs. 2 months), although this difference was not significant (P = 0.057). (**B**) The median overall survival (OS) in the CIT ≥ 3 courses group was significantly longer than that of patients in the CIT < 3 courses group (23 vs. 9 months; P = 0.020)

In the multivariate analysis, we found that sex, age, smoking history, ECOG performance status, chemotherapy courses, chemotherapy responses, radiotherapy and surgery had no effect on the prognosis of SCLC patients receiving CIT (P > 0.05). We then investigated the influence of the CIT frequency on the prognosis of patients in the study group, in which the median frequency of CIT was 3 courses (range, 1–8 courses). Patients were divided into two subgroups: CIT ≥ 3 courses and < 3 courses. The characteristics of the patients in these subgroups were well balanced (Table 3). The median PFS in the CIT ≥ 3 courses group (n = 17) was longer than that of the CIT < 3 courses group (n = 12) (9.5 vs. 2 months), but this difference was not significant (P = 0.057) (HR, 0.465; 95 % CI, 0.204–1.063; P = 0.070) (Fig. 4A). However, the median OS of the CIT ≥ 3 courses group was significantly longer than that of the CIT < 3 courses group (23 vs. 9 months, P = 0.020; HR, 0.335, 95 % CI, 0.125–0.893, P = 0.029) (Fig. 4B).

**Table 3:** Clinical characteristics of patients who received CIT for ≥ 3 courses or < 3 courses

Clinical features	CIT ≥ 3 courses	CIT < 3 courses	P-value
Sex			
Male	11	11	0.187
Female	6	1	
Age, years			
Median (range)	61 (41–79)	64 (54–78)	0.107
Smoking index			
Median (range)	400 (0–1000)	350 (0–2000)	0.751
ECOG			
≤1	16	9	0.279
2	1	3	
Chemotherapy courses			
Median	6	6	0.713
Range	4–6	4–6	
Radiotherapy			
Yes	16	9	0.279
No	1	3	
Surgery			
Yes	3	1	0.622
No	14	11	
Chemotherapy responses *			
CR	5	2	0.707
PR	7	7	
SD	5	3	
Stage			
Limited	10	7	1.000
Extensive	7	5	

Abbreviations: CIT, cellular immunotherapy; ECOG, Eastern Cooperative Oncology Group; CR, complete remission; PR, partial remission; SD, stable disease

Note: *The responses for the first-line therapy

### Response to second-line chemotherapy

A total of 18 patients accepted second-line therapy in the study group, and 17 patients received second-line therapy in the control group. The median second-line chemotherapy courses in the study and control group were 3 (range, 1–6) and 2 (range, 1–6), respectively, but there was no significant difference between two groups (P > 0.05). None of the patients in two groups achieved a CR after second-line treatment. Eight patients got a PR or SD with a CBR of 44.4 % in the study group, and 5 patients got a PR or SD with a CBR of 29.4 % in the control group, but there was no significant difference between the groups (P = 0.489).

### Side effects of CIT infusion

Two patients reported mild fatigue after CIT infusion. One patient had a transient fever of 37.8 °C after one infusion, but recovered after 1 hr. No other significant side effects were observed.

### Proportion of the immune cells in peripheral blood before and after CIT

The proportion of T, NK, NKT, B, Treg cells and monocytes were analyzed before and after CIT. However, there was no significant change in these cells. There were relatively less Treg cells at some time points after CIT, but these differences were not significant (data not shown).

## Discussion

Immune escape in SCLC patients is closely associated with the recurrence of the disease, and may contribute to poor patient survival [8, 9, 12–14]. However, the findings of several studies suggest that maintenance and/or consolidation using cytokines and other biological agents fail to improve SCLC outcomes [7]. The therapeutic effects of CIT in SCLC have rarely been reported in either preclinical or clinical studies. Meanwhile, cancers employ multiple mechanisms to evade an immune response [29–31], and thus CIT with only one type of immune cells is unlikely to achieve an optimal anti-cancer effect. NK and γδT cells were shown to have a synergistic anti-cancer effect in a preclinical study [22]. Besides, chemotherapy could not only alleviate immune suppression by reducing the tumor burden [32, 33], but also up-regulate the expression of the NKG2D ligands on cancer cells, thus sensitizing them to lysis mediated by NKG2D-expressing lymphocytes [34]. In this study, we applied CIT with a combination of NK, γδT and CIK cells as maintenance therapy after systemic chemotherapy, and showed survival advantage for SCLC patients for the first time. At the same time, another study conducted by our group also confirmed the efficacy of the CIT with combination of these immunocytes in improving the outcome of patients with hepatocellular carcinoma after radiofrequency ablation (RFA) [25]. Thus, CIT may be a novel treatment for SCLC patients who respond to first-line therapy, and could provide a novel strategy for the devastating disease.

OS is widely used as a primary end point for immunotherapy in many cancers, such as melanoma [35, 36] and prostate cancer [37]. SCLC is a highly malignant tumor associated with short survival and limited chemotherapy regimens, and hence there are less confounding factors in the observation of OS. In our study, OS was significantly longer after CIT both in LS-SCLC and ES-SCLC patients. There are several possible reasons for the prolonged survival of SCLC patients with CIT. Cancer stem cells (CSCs) have been reported to be responsible for cancer progression, metastasis, and the development of drug resistance [38, 39]. They have also been shown to be susceptible to immunocyte-mediated toxicity, suggesting that CIT may be useful in eliminating minimal residual disease (MRD) and thus reducing cancer recurrence [40–42], which may explain why CIT maintenance therapy prolongs PFS and consequently prolongs survival of SCLC patients. Although PFS has recently been shown to be a good predictor of OS in SCLC [43], but PFS did not differ significantly between the groups in LS-SCLC patients. It is noteworthy though that another study exploring the relationship between PFS and OS in SCLC found no significant relationship between them [44], suggesting that OS might be affected by other factors, such as second or third-line therapy.

It has been reported that patients who receive cancer vaccines respond better to subsequent chemotherapy than those who do not, suggesting that immunotherapy might sensitize cancer cells to cytotoxic drugs [45, 46]. The CBR of the relapsed patients to second-line treatment was also detected in our study. Patients with CIT tended to have higher CBR than the control, although there was no significant difference between two groups, indicating that CIT might enhance the sensitivity of recurrent SCLC to second-line chemotherapy. This is a potential mechanism by which OS could be extended by CIT, although the exact mechanistic basis for this needs to be further explored.

The quality of the cultured immunocytes is of primary importance in CIT. Both the purity and viability of the immune cells in our study were acceptable, and indeed they were actually better than those in previously reported expansion methods [47, 48]. The percentage of activated NK cells (CD56^+^CD69^+^) was also significantly increased after induction, indicating that the activity of NK cells was remarkably improved. Each kind of immunocytes had strong cytotoxicity when used alone, but the highest cytotoxic effect was achieved when they were combined, reflecting a strong synergy between these cells. Safety is another important factor determining the application of CIT. We did not observe any significant side effects after CIT, and only 2 patients felt mild fatigue and 1 patient had a transient fever during 1 of the infusions. All the symptoms diminished without treatment. Thus, CIT was well tolerated in our study, and it may be a good candidate for maintenance therapy when side effects need to be minimized.

Potential factors influencing the outcome of CIT were also detected in this study. Using a multivariate analysis, we found that sex, age, smoking history, ECOG performance status, chemotherapy courses, chemotherapy responses, radiotherapy and surgery were not related to the efficacy of treatment. However, the OS of patients who received more than 3 courses of CIT was significantly longer than that of patients who received less than 3 courses of CIT. PFS also tended to be longer in patients who received more than 3 CIT courses, although this was not statistically significant. It was previously demonstrated that NSCLC patients who received more than 7 courses of CIK cell treatment had a significantly better prognosis than those who received fewer courses [49]. Taken together, our findings and those of a previous report [49] suggest that a greater number of CIT courses improves patient outcome. However, the optimal number of treatment courses and the duration of treatment are yet to be determined. Besides, bias might exist in the analysis of the influence of the CIT frequency on the prognosis of patients. Even though the characteristics of the patients in these subgroups were well balanced, some patients stopped CIT treatment in CIT < 3 courses group because of disease progression. Therefore, the shorter PFS and OS might be affected not only by less frequency of CIT treatment, but also by other factors, such as the worse features of the disease status in this subgroup of patients. However, these results could provide an instructive reference for subsequent clinical trials.

There is no consensus on the best indicators for assessing immune response after CIT. In this study, T, NK, NKT, B, Treg cells and monocytes populations in the peripheral blood were analyzed before and after CIT. However, there was no significant change in these cells. There were relatively less Treg cells at some time points after CIT, but these differences were not significant. This might reflect the relatively few CIT courses and the small number of patients involved. However, it may also be that the proportion of immune cells in the peripheral blood does not relate to the response to CIT, and this needs to be addressed in a further study.

CIT could improve the quality of life of cancer patients as reported [50]. However, the patients enrolled in this study had a good performance status (ECOG 0–2) before disease progression, and the status of the patients showed no significant difference between the 2 groups. Therefore, it was difficult to determine whether CIT affected the quality of life in this study.

In our study, ES-SCLC patients who underwent CIT were on average older than those who did not undergo therapy. However, the multivariate analysis showed that age had no effect on the prognosis of these patients. It has also been reported that effector cell function decreases in older people, and might be associated with reduced antitumor immunity in these patients [51]. Additionally, patients in the study group had longer PFS and OS, indicating that CIT may effectively reduce immunosuppression, provide an anti-cancer effect and prolong OS, even in elderly patients.

This study was designed as a prospective cohort study. The patients were enrolled to each group of the study based on their choice of the therapeutic options. Therefore, this design allowed us to collect valuable data on the efficacy of CIT, and better reflects a practical clinical settings. At the same time, although innovative cancer treatments might bring benefits to the cancer patients, cost effectiveness is an important aspect that must be considered in the clinical practice. Nowadays, maintenance therapy for SCLC is very limited. CIT as a maintenance therapy has shown its potential to prevent disease recurrence and prolong the survival of SCLC patients with minimal side effects in our study. Besides, the methods of the preparation of immune cells are very simple compared with the complicated preparation methods of other CIT therapies, such as sipuleucel-T, which is used in prostatic cancer and chimeric antigen receptor T-cell immunotherapy (CAR-T) applied in B cell leukemia. And its price might be no more than those of other available maintenance therapies for NSCLC such as erlotinib, bevacizumab and pemetrexate. Therefore, CIT as maintenance therapy may be a novel cost effective strategy for SCLC therapy, and warrants further investigation.

## Conclusions

CIT as a maintenance therapy after first-line treatment is well tolerated, and it might prevent disease recurrence and prolong the survival of SCLC patients. We also found that more frequent CIT administration might improve treatment outcome. The exact mechanism through which CIT extends PFS and OS in SCLC patients remains unclear and a multi-center randomized clinical trial is needed to further verify its efficacy.

## Additional files

Additional file 1: Table S1. The number of the 3 types of immunocytes infused into each patient. Additional file 2: Figure S1: The cytotoxic effect of immune cells on NCI-H446 cells. All 3 types of immune cells exhibited a significant cytotoxic effect on NCI-H446 cells when used alone, but a greater cytotoxic effect was achieved when they were combined, and this cytotoxicity increased further as the E/T cell ratio increased. At an E/T cell ratio of 25:1, the median cytotoxicity level of NK, γδT and CIK cells was 75.5 % (range, 59.1–90.7 %), 70.6 % (range, 48.3–80.0 %), and 49.0 % (range, 27.4–68.9 %), respectively. The combined immune cells showed a synergistic anti-cancer effect with a median cytotoxicity level of 85.5 % (range, 63.4–96.5 %) at an E/T cell ratio of 25:1.

## Abbreviations

CBR: Clinical benefit rate
CI: Confidence interval
CIK: Cytokine-induced killer
CIT: Cellular immunotherapy
CR: Complete remission
CT: Computed tomography
CSCs: Cancer stem cells
CTL: Cytotoxic T lymphocyte
CTLA-4: Cytotoxic T-lymphocyte antigen-4
EC: Etoposide/carboplatin
ECOG: Eastern Cooperative Oncology Group
EP: Etoposide/cisplatin
ES-SCLC: Extensive stage small cell lung cancer
GMP: Good Manufacturing Practice
HR: Hazard ratio
irPFS: immune-related progression free survival
LS-SCLC: Limited stage small cell lung cancer
LDH: Lactate dehydrogenase
MRD: Minimal residual disease
MRI: Magnetic resonance imaging
NK: Natural killer
NSCLC: Non-small cell lung cancer
OS: Overall survival
PBMCs: Peripheral blood mononuclear cells
PD: Progressive disease
PFS: Progression free survival
PR: Partial remission
RFA: Radiofrequency ablation
SCLC: Small cell lung cancer
SD: Stable disease
SOP: Standard operating procedure
