# Supplementary material for: Cellular immunotherapy as maintenance therapy prolongs the survival of the patients with small cell lung cancer
Source: J Transl Med. 2015 May 13;13:158. doi: 10.1186/s12967-015-0514-0 (PMC4446113; doi:10.1186/s12967-015-0514-0)
Supplement: Additional file 1: Table S1. — The number of the 3 types of immunocytes infused into each patient. [file 12967_2015_514_MOESM1_ESM.pdf]

Table s1. The number of the 3 types of immunocytes infused into each patient.

Patient	CIT Courses	NK cells			γδT cells			CIK cells		
		Median* (×10^9)	Range (×10^9)	Number of treatments	Median* (×10^9)	Range (×10^9)	Number of treatments	Median* (×10^9)	Range (×10^9)	Number of treatments
1	3	1.6	1.4–1.9	12	1.3	1.2–1.5	6	1.6	1.2–2.0	18
2	2	1.4	1.2–1.8	8	1.3	1.2–1.5	4	1.5	1.3–1.8	12
3	5	1.4	1.2–1.7	20	1.4	1.3–1.5	10	1.5	1.3–2.0	30
4	1	1.3	1.2–1.5	4	1.3	1.2–1.4	2	1.4	1.2–1.5	6
5	2	1.3	1.2–1.4	8	1.3	1.2–1.5	4	1.4	1.2–1.7	12
6	1	1.5	1.2–1.8	4	1.3	1.2–1.4	2	1.3	1.2–1.5	6
7	3	1.6	1.3–2.0	12	1.4	1.2–1.6	6	1.6	1.3–2.0	18
8	7	1.5	1.2–1.8	28	1.4	1.2–1.5	14	1.5	1.2–1.9	42
9	4	1.7	1.4–2.0	16	1.4	1.2–1.9	8	1.6	1.2–2.0	24
10	2	1.4	1.2–1.8	8	1.4	1.3–1.6	4	1.4	1.2–1.7	12
11	1	1.3	1.2–1.4	4	1.4	1.2–1.6	2	1.3	1.2–1.5	6
12	6	1.5	1.2–1.7	24	1.5	1.3–1.7	12	1.6	1.2–2.0	36
13	8	1.4	1.2–1.9	32	1.4	1.2–1.7	16	1.5	1.2–1.8	48
14	1	1.6	1.5–1.7	4	1.4	1.3–1.5	2	1.4	1.2–1.6	6
15	4	1.5	1.2–2.0	16	1.5	1.2–1.7	8	1.6	1.3–2.0	24
16	3	1.5	1.3–1.7	12	1.4	1.2–1.7	6	1.5	1.3–1.7	18
17	8	1.4	1.2–1.8	32	1.3	1.2–1.5	16	1.5	1.2–2.0	48
18	6	1.5	1.2–1.7	24	1.4	1.2–1.7	12	1.5	1.2–1.8	36
19	7	1.6	1.3–1.9	28	1.4	1.2–1.7	14	1.5	1.2–2.0	42
20	5	1.6	1.2–1.8	20	1.4	1.2–1.6	10	1.6	1.2–2.0	30
21	2	1.4	1.3–1.5	8	1.2	1.2–1.3	4	1.4	1.2–1.6	12
22	8	1.5	1.2–2.0	32	1.5	1.2–1.8	16	1.7	1.2–2.0	48
23	3	1.4	1.2–1.7	12	1.4	1.2–1.7	6	1.5	1.2–1.7	18
24	2	1.3	1.2–1.6	8	1.3	1.2–1.5	4	1.3	1.2–1.5	12
25	2	1.4	1.2–1.6	8	1.4	1.2–1.5	4	1.4	1.2–1.7	12
26	2	1.5	1.4–1.7	8	1.4	1.3–1.5	4	1.5	1.3–1.7	12
27	2	1.5	1.2–1.9	8	1.4	1.2–1.8	4	1.4	1.2–1.9	12
28	7	1.6	1.4–2.0	28	1.6	1.2–1.9	14	1.7	1.4–2.0	42
29	6	1.5	1.2–1.7	24	1.4	1.2–1.7	12	1.6	1.3–2.0	36

Abbreviations: CIT, cellular immunotherapy; NK, natural killer; CIK, cytokine-induced killer.

Note: * The median number of each type of the immunocytes for each infusion.
