# Supplementary figures and images for: Cellular immunotherapy as maintenance therapy prolongs the survival of the patients with small cell lung cancer
Source: J Transl Med. 2015 May 13;13:158. doi: 10.1186/s12967-015-0514-0 (PMC4446113; doi:10.1186/s12967-015-0514-0)

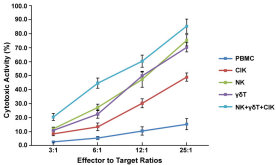

Supplement: Additional file 2: Figure S1: — The cytotoxic effect of immune cells on NCI-H446 cells. All 3 types of immune cells exhibited a significant cytotoxic effect on NCI-H446 cells when used alone, but a greater cytotoxic effect was achieved when they were combined, and this cytotoxicity increased further as the E/T cell ratio increased. At an E/T cell ratio of 25:1, the median cytotoxicity level of NK, γδT and CIK cells was 75.5 % (range, 59.1–90.7 %), 70.6 % (range, 48.3–80.0 %), and 49.0 % (range, 27.4–68.9 %), respectively. The combined immune cells showed a synergistic anti-cancer effect with a median cytotoxicity level of 85.5 % (range, 63.4–96.5 %) at an E/T cell ratio of 25:1. [file 12967_2015_514_MOESM2_ESM.pdf]
